# Critical Dynamics of Gravito-Convective Mixing in Geological Carbon Sequestration

**DOI:** 10.1038/srep35921

**Published:** 2016-11-03

**Authors:** Mohamad Reza Soltanian, Mohammad Amin Amooie, Zhenxue Dai, David Cole, Joachim Moortgat

**Affiliations:** 1 School of Earth Sciences, the Ohio State University, Columbus, Ohio, USA; 2 Los Alamos National Laboratory, Los Alamos, New Mexico, United States

## Abstract

When CO_2_ is injected in saline aquifers, dissolution causes a local increase in brine density that can cause Rayleigh-Taylor-type gravitational instabilities. Depending on the Rayleigh number, density-driven flow may mix dissolved CO_2_ throughout the aquifer at fast advective time-scales through convective mixing. Heterogeneity can impact density-driven flow to different degrees. Zones with low effective vertical permeability may suppress fingering and reduce vertical spreading, while potentially increasing transverse mixing. In more complex heterogeneity, arising from the spatial organization of sedimentary facies, finger propagation is reduced in low permeability facies, but may be enhanced through more permeable facies. The connectivity of facies is critical in determining the large-scale transport of CO_2_-rich brine. We perform high-resolution finite element simulations of advection-diffusion transport of CO_2_ with a focus on facies-based bimodal heterogeneity. Permeability fields are generated by a Markov Chain approach, which represent facies architecture by commonly observed characteristics such as volume fractions. CO_2_ dissolution and phase behavior are modeled with the cubic-plus-association equation-of-state. Our results show that the organization of high-permeability facies and their connectivity control the dynamics of gravitationally unstable flow. We discover new flow regimes in both homogeneous and heterogeneous media and present quantitative scaling relations for their temporal evolution.

Geological sequestration of carbon dioxide (CO_2_) has been proposed as a technology to reduce the amount of CO_2_ emitted into the atmosphere^1^,^2^,^3^,^4^,^5^,^6^. The main processes controlling the trapping of CO_2_ during geological sequestration are storage of supercritical CO_2_ in a gas cap, CO_2_ dissolution in brine, known as solubility trapping^7^,^8^, residual trapping due to hysteresis^9^,^10^, and mineral trapping by CO_2_ precipitation as secondary carbonates^11^. In this work, we focus on solubility trapping, which can be enhanced when gravitational instabilities (fingering) are triggered by a local increase in brine density as CO_2_ dissolves into brine in the top of an aquifer. This Rayleigh-Taylor-type instability^12^ is sometimes referred to as *gravito-convective mixing* and involves both diffusive and advective motion of dissolved CO_2_, with advection being the dominant driving force. Whether the interface between CO_2_-bearing brine and fresh brine becomes gravitationally unstable depends on the ratio of advection to molecular diffusion (Rayleigh or Péclet numbers). When a fingering instability is triggered, dissolved CO_2_ is mixed throughout the aquifer at *advective* time-scales, which can be much faster than diffusive transport^12^,^13^,^14^. This improves the storage capacity of a given aquifer and decreases the leakage risk in case of cap rock failure.

There is extensive literature on gravitational instabilities^14^,^15^,^16^,^17^,^18^. Stability analyses and numerical simulations predict a critical wavelength for the instability that depends on fluid and reservoir properties as^13^,^14^,^15^,^16^,^17^,^18^,^19^:


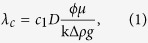


with brine viscosity, *μ*, porosity, *ϕ*, CO_2_ diffusion coefficient, *D*, permeability, k, maximum density increase of the aqueous phase upon CO_2_ dissolution, Δ*ρ*, and gravitational acceleration, *g*. A critical onset time is predicted by:


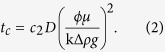


Different proportionality constants *c*_1_ and *c*_2_ have been obtained by the different authors cited above.

It is evident from Eq. (1) and Eq. (2) that the critical wavelength and onset time depend on porosity and permeability. Since the spatial variability of porosity is much smaller than that of permeability, the latter is often used to represent the heterogeneity of porous media^20^,^21^,^22^.

Various studies have characterized the transport of CO_2_-rich brine through heterogeneous systems. Using numerical simulations, Farajzadeh *et al*.^23^ studied the influence of spatial heterogeneity in permeability on gravitational fingering, and explained three different flow regimes for stable trapping of dissolved CO_2_: fingering, dispersion, and channeling. Different models of permeability heterogeneity have been tested by previous authors^24^,^25^. These studies concluded that permeability heterogeneity could have a significant impact on the onset, rate, and characteristic behavior of density-driven flow of CO_2_. On the other hand, theoretical^26^, numerical^27^, and experimental^6^ results suggest that density-driven flow may not always lead to significant convective mixing especially in layered systems containing low-permeability zones; CO_2_ may actually become immobilized in these zones. In order to quantify the effects of vertical heterogeneity, Green and Ennis-King^28^ developed a simple model consisting of a *random* and *uncorrelated* distribution of horizontal, impermeable barriers with a given overall volume fraction (0.04% and 0.15%). They found that in a homogeneous medium with equivalent effective vertical permeability, compared to heterogeneous medium, convection begins at later onset times.

In reality, geological heterogeneity is controlled by the spatial organization of sedimentary facies with different physical attributes, such as permeability^29^. Facies distributions therefore affect species transport through a formation^9^. Architectures of sedimentary facies can exhibit sharp discontinuities across boundaries between depositional features, for instance across the sandstone-shale contacts that are common in fluvial deposits. The complex patterns in such fluvial architectures^30^ exist in many candidate aquifers for CO_2_ sequestration^9^,^31^,^32^ and result in variable connectivity and *tortuous* flow pathways^33^. Representing such discontinuous, *correlated* heterogeneity in reservoir simulations is non-trivial^34^, and its influence on the characteristics of density-driven flow has not been studied in detail. In this work, we generate many volume-preserving realizations of bimodal facies architectures using a Markov Chain approach combined with an indicator simulation with quenching. For comparison, homogeneous domains are defined by the equivalent geometric mean permeability for each realization. We then perform high-resolution two-dimensional simulations of gravitational fingering in both homogeneous and heterogeneous media to investigate the influence of facies-based heterogeneity and connectivity on the transport of dissolved CO_2_.

For accurate numerical simulations of gravitational fingering, the physics of fluid flow and thermodynamics have to be represented rigorously. High-resolution numerical discretizations are required to minimize numerical dispersion which can obscure the onset of small-scale fingers. We adopt a combination of the mixed hybrid finite element (MHFE) method –to simultaneously solve for continuous pressure and velocity fields– and a higher-order discontinuous Galerkin (DG) method for the species transport. Phase behavior is described by the cubic-plus-association (CPA) equation of state (EOS). CPA-EOS has been shown to accurately reproduce measured densities of CO_2_-brine mixtures and the *volume swelling* due to CO_2_ dissolution^1^,^35^,^36^. Volume swelling and the associated movement of the interface between free CO_2_ in a gas cap and the underlying brine, cannot be modeled by considering only the aqueous phase^15^ and replacing the gas cap with a constant-CO_2_-composition Dirichlet boundary condition^37^. In this work, we *inject* CO_2_ from the upper boundary at a sufficiently low rate that the simulated domain remains a single aqueous phase (i.e., without a capillary transition zone^22^). We do *not* adopt the Boussinesq approximation in order to fully study the effects of fluid compressibility and the pressure response due to swelling effect when increasing volumes of CO_2_ are injected in a confined domain (impermeable boundaries) and subsequently dissolve in the brine^1^,^18^.

## Numerical Experiments

We consider CO_2_ sequestration in saline aquifers by injecting CO_2_ uniformly from the top without production of brine (alternatively, this can be interpreted as a CO_2_ dissolution rate from an overlying CO_2_-gas cap). The rate is kept sufficiently low (0.1% pore volume (PV) per year) to insure that brine is nearly CO_2_-saturated in the top but without forming a gas phase in the computational domain. Because saline aquifers are large, injection of 1–2% PV in 10–20 years is sufficient, and higher injection volumes result in excessive pressure increases. We model a 12 m × 30 m subdomain, discretized by a fine 240 × 300 grid and with a uniform (or heterogeneous average) porosity of 10%. The constant temperature is 77 °C and the initial pressure is 100 bar at the bottom. At these conditions, the pure and CO_2_-saturated brine densities are *ρ*_*w*_ = 978 kg/m^3^ and *ρ* = 978 kg/m^3^, with Δ*ρ* = (*ρ*−*ρ*_*w*_)/*ρ*_*w*_ = 0.9%, for a CO_2_ solubility of 1.7 mol%. The diffusion coefficient is *D* = 1.33 × 10^−8^ m^2^ s^−1^.

**Figure 1:**
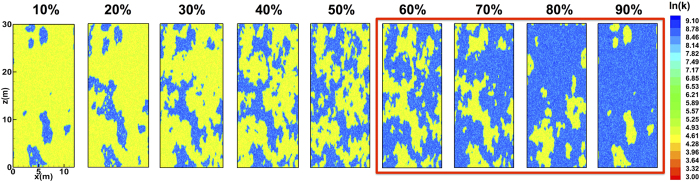
Bimodal heterogeneous permeability fields with volume fraction of high-permeability facies (blue color) varying from 10% to 90%. The architectures in the red box have fully connected high-permeability pathways spanning opposing boundaries of the domain.

Whether spatial heterogeneity of an aquifer enhances density-driven flow of CO_2_-rich brine depends on the volume fraction of high-permeability facies and their connectivity. Our long-term goal is to perform large two- and three-dimensional simulations with multiple facies types. In this preliminary study we show the fundamental importance of heterogeneities arising from facies architecture. We choose a parsimonious set of two facies types representing a bimodal architecture^29^,^38^. Different structures are created using a Markov Chain model and indicator simulation with quenching in TPROGS^39^. Rather than focusing on any single site or data set, we represent general permeability distributions consisting of high- and low-permeability facies. The high-permeability facies volume fraction is increased from 10% to 90% in increments of 10% (Fig. 1), which also increases the facies connectivity^29^,^40^. In all cases, the mean length of the less abundant facies are 2 m in both horizontal and vertical directions representative of meter-scale sedimentary features. For each volume fraction, we generate five realizations of facies structures and for each realization we superimpose a lognormal permeability distribution with a variance of 0.1 within each facies. The geometric mean of high and low-permeability facies are 8.5 (5,000 md) and 4.6 (100 md), respectively^29^. For comparison, we create homogeneous domains with the same mean permeabilities (Table 1).

## Results and Discussions

To characterize density-driven flow of CO_2_, we use the method from a seminal paper by Sudicky^41^. The spatial moments of the CO_2_ distribution provide an insightful measure for the transport of CO_2_ at various time-scales. The first three central moments of the distribution are employed to define the spatial variance, 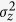, of the CO_2_ molar density as an indicator of vertical *spreading* (dispersion) and a reasonable proxy to mixing. The square root of spatial variance, *σ*_*z*_, is defined as the dispersion-width (scale) to determine the extent to which the descending plume of CO_2_ has stretched (i.e., the volume of CO_2_-enriched brine). These definitions are in line with models developed for flow and transport in systems having complex spatial variability in physical and chemical properties^42^,^43^, but also take into account the density change due to dissolution and compressibility. In our analyses, we consider the dynamics from the first contact of CO_2_ with brine until 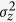 or *σ*_*z*_ reach their maximum values –determined by domain dimensions– above which they plateau (see *Methods*). Next, we study the temporal evolution of 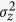 and note that faster vertical motion implies reaching the maximum variance in a shorter amount of time (Eq. (7)). Fast mixing and spreading towards higher depths improve the long-term storage of dissolved CO_2_ throughout the aquifer^6^, because CO_2_ is likely to remain in aquifer due to residual and mineral trapping even if the pressure were to drop due to a failure of the cap rock.

**Figure 2:**
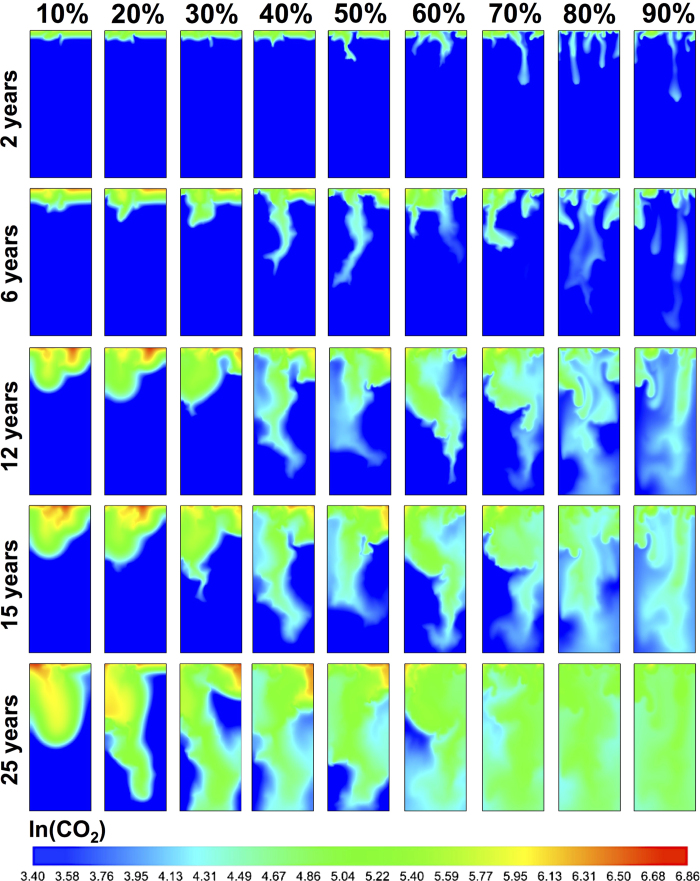
Natural log of molar density of CO_2_ after 2, 6, 12, 15, and 25 years for bimodal heterogeneous domains (for the realizations shown in Fig. 1).

**Figure 3:**
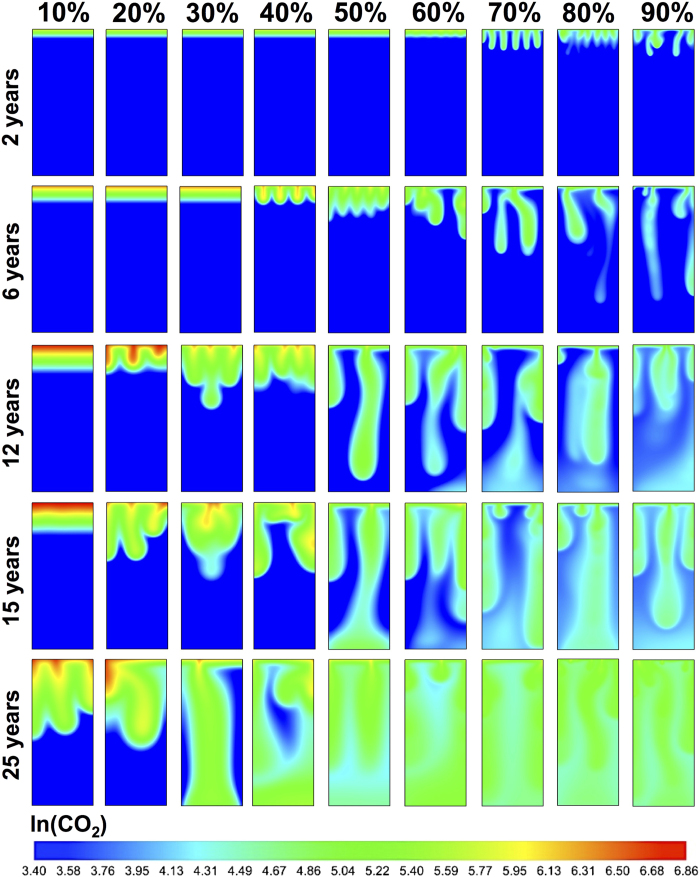
Natural log of molar density of CO_2_ after 2, 6, 12, 15, and 25 years for homogeneous domains.

**Figure 4:**
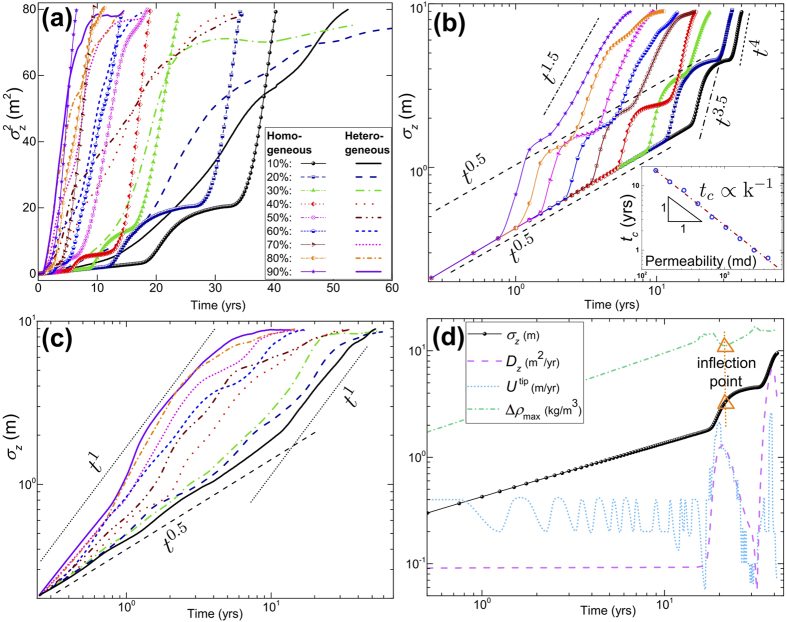
(a) Spatial variance of molar density of CO_2_ (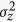) for all homogeneous and ensemble (i.e., averaged over 5 realizations) heterogeneous cases. **(b)** Dispersion-width of molar density of CO_2_ (*σ*_*z*_) for homogeneous domains. **(c)** Ensemble *σ*_*z*_ for heterogeneous cases. **(d)** Dispersion coefficient, defined as 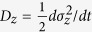, and tip velocity of the most advanced finger, defined as *U*^ tip^ = *dz*^tip^/*dt* where *z*^tip^ is the tip vertical depth, for one homogeneous domain corresponding to 10% of the high-permeability facies (initial fluctuations of tip velocity are of order Δ*z*/Δ*t* due to the numerical differentiation of tip location with respect to time). Onset of different regimes and critical features correlate well with each other through different measurements of *σ*_*z*_, *D*_*z*_, *U*^ tip^, and the maximum Δ*ρ* (see Fig. 5 for global scaling of the latter).

**Figure 5:**
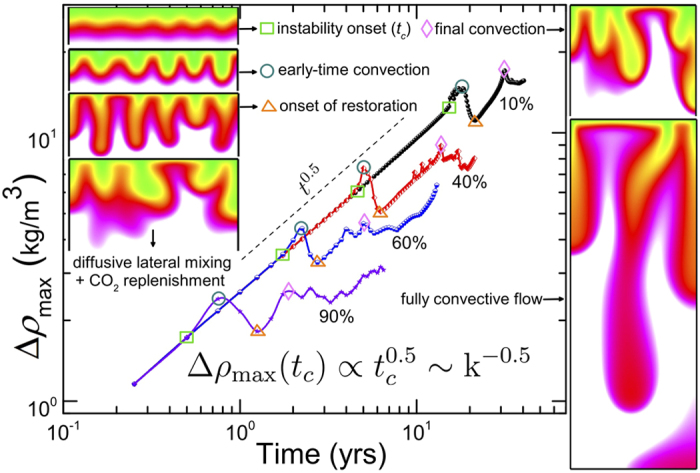
Mass density difference between fresh brine and *partially-saturated* brine at top boundary, i.e. the maximum Δ*ρ*, for homogeneous media. Results are shown for 4 different geometric mean permeabilities equivalent to 10, 40, 60, and 90% volume fractions of high-permeability facies. Visualizations of the 60% case illustrate universal features revealed by this analysis. The onset of various regimes seen in the spatial variance of CO_2_ can be understood by variations in the *driving force*. In addition, a consistent relation is obtained between critical onset time of the instability, geometric mean permeability, and maximum Δ*ρ* at the onset, which also satisfies the traditional scaling of Eq. (2). Panels in Fig. 3 corresponding to these different flow regimes are discussed in the text.

We summarize our main findings in Figs 2 and 3, which show the molar density of dissolved CO_2_ at 2, 6, 12, 15, and 25 years for heterogeneous media and their homogeneous equivalents. Density-driven flow in homogeneous domains has been extensively studied before, though with simplifying assumptions in terms of boundary conditions and phase behavior. Phase behavior is critical because the *driving force* for gravitational instabilities is the density contrast, Δ*ρ*, upon CO_2_ dissolution. From our scaling analyses (Fig. 4) for fully compositional EOS-based simulations of compressible flow, we observe new flow regimes even for homogeneous domains due to the non-trivial evolution of maximum Δ*ρ* over time (Fig. 5). In the following, we first discuss these results for homogeneous media and then for different bimodal heterogeneous facies architectures.

### Homogeneous media

We observe four distinct flow regimes in all simulations for homogeneous media, but most clearly identifiable at low mean permeabilities (Fig. 4a,b). Dependencies on the mean formation permeability can be related to the Rayleigh number, which is the ratio of buoyancy to diffusive flux: *Ra* = k*g*Δ*ρH*/*μϕD*, with *H* the depth of the domain^44^. In this study, the Rayleigh number is equivalent to the Péclet number because the advective Darcy flux is predominantly gravity-driven.

Traditionally, a constant pressure and CO_2_-composition are assumed at the top boundary of a brine-saturated subdomain. Consequently, the density contrast between pure and CO_2_-saturated brine in the top remains at a *constant*, maximum value Δ*ρ*_max_, which has been used to estimate *t*_*c*_, *λ*_*c*_, and *Ra*^15^. For a nearly constant brine viscosity and CO_2_ diffusion coefficient, the critical wavelength and time only depend on rock properties k and *ϕ*. In our work, we continuously inject CO_2_ and the composition in the top depends both on the injection rate, *and* on the downward transport rate of dissolved CO_2_. The latter also depends on permeability: for low permeabilities, buoyancy-driven Darcy fluxes are slower and more injected CO_2_ accumulates in the top. This gradually increases the brine density and makes the dissolution front increasingly unstable (positive feedback). Conversely, for high permeabilities, downward transport of dissolved CO_2_ is faster, which decreases the CO_2_ composition and thus the unstable density-contrast in the top (negative feedback). Swelling, and the associated pressure response, due to different rates of CO_2_ dissolution further complicate the evolution of Δ*ρ*_max_. These different feedbacks between transport dynamics and the buoyant driving force result in the four flow regimes (Figs 4 and 5) that we discuss in the next paragraphs.

1. Initially, CO_2_ transport is controlled by diffusion as long as the CO_2_-front remains stable and advective flow is less than the diffusive flux (with small Δ*ρ*_max_ resulting in low Rayleigh/Péclet numbers). Molecular diffusion involves low dissolution and mixing rates. The front travels to a ‘diffusion penetration depth’ which obeys a ~(*Dt*)^0.5^ Einstein-type scaling^45^. Likewise, the dispersion-width shows a classical Fickian scaling of *σ*_*z*_ ∝ *t*^0.5^ in Fig. 4b. We also keep track of the vertical propagation speed of the most advanced finger tip, which is shown in Fig. 4d for one case. The tip velocities are consistent with our measure of the dispersion coefficient (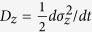). This first flow regime is observed in all homogeneous cases and as long as Rayleigh numbers are small, diffusion-dominated transport is insensitive to permeability (and for nearly constant *μ* and *D* and small Δ*ρ*_max_, also independent of *Ra*). Interestingly, while the total amount of introduced CO_2_ increases linearly with time (due to constant injection rate), the driver for *advective* buoyant flow, Δ*ρ*_max_, also follows Fickian ~*t*^0.5^ scaling until it has increased sufficiently to trigger gravitational instabilities (Fig. 5). The onset time, *t*_*c*_, of this next flow regime *does* depend on permeability (and *Ra*). The instability onset for the 60% volume fraction can also be observed in Fig. 3 after 2 years.
2. The onset of fingering can be recognized as the transition to a *convection-dominated* regime^15^ (high Rayleigh numbers), which manifests itself as a sharp increase in 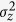 and *σ*_*z*_ (Fig. 4). This regime occurs after ~6 years in Fig. 3 for the 40% volume fraction. Table 1 summarizes the critical times from stability analysis^13^,^14^,^19^,^46^ as well as the critical times derived from the deviation of *σ*_*z*_ from Fickian scaling (inset of Fig. 4b). It has been recognized that the initial exponential growth of a dominant harmonic perturbation, as predicted by linear stability analyses, may not necessarily be apparent in numerical simulations or even in experiments. Observed *t*_*c*_ are generally higher by at least an order of magnitude^1^,^14^. As discussed above, Eq. (2) also does not fully take into account the non-linear evolution of Δ*ρ*_max_. The dependence of critical times and wavelengths on permeability (Eqs (1)–(2), 2 and Table 1) imply that higher permeabilities result in an earlier onset time with a larger number of small-scale fingers, illustrated in Fig. 3. However, Fig. 4b shows a new scaling relation of *t*_*c*_ ∝ *k*^−1^, rather than the *t*_*c*_ ∝ *k*^−2^ from stability analyses in Eq. (2). This is because Δ*ρ*_max_ itself evolves. Since Δ*ρ*_max_(*t*) ∝ *t*^0.5^ in the first regime (Fig. 5), we find that Δ*ρ*_max_(*t*) ∝ k^−0.5^. Using this scaling of Δ*ρ*_max_ in Eq. (2) is consistent with our independent finding for the scaling of *t*_*c*_ with k.A second interesting new finding is that the initial scaling of the dispersion-width in the fingering regime is *σ*_*z*_ ∝ *t*^3.5^ for *all* permeabilities. This is comparable to the Rayleigh-independent convective mixing rate found in Hidalgo *et al*.^12^. We argue that this independence from Rayleigh number is because 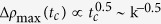, and the critical time *t*_*c*_ in Eq. (2) is related to a Rayleigh number in which *H* is the thickness of the propagating diffusive layer before the onset of instabilities^47^, rather than the domain height. Because the initial diffusive layer grows as *t*^0.5^, Δ*ρ*_max_(*t*_*c*_) × *H*(*t*_*c*_) ∝ *t*_*c*_ ~ k^−5^ and the Rayleigh number at the onset of fingering (*Ra*(*t*_*c*_) ∝ kΔ*ρ*_max_*H*) becomes insensitive to permeability.
3. We observe a third flow regime that has not been clearly recognized in the literature before. Figure 4 shows an inflection point in the spatial variance curves, followed by another regime of diffusion-type scaling. This regime can be seen in Fig. 3 for a volume fraction of 10% after 25 years. The cause of this diffusive regime is three-fold. First, persistent coalescence and merging of growing fingers involve lateral diffusion, which result in coarsened fingers. Second, as the fingers *stretch*, the compositional gradients across the finger surface become steeper due to compression, which enhances diffusive fluxes across the finger boundaries^48^. Third, diffusion is further promoted by the nature of Rayleigh-Taylor fluid dynamics: downward motion of (growing) dense fingers and subsequent upwards displacement of fresh lighter brine increase the surface area of the CO_2_ dissolution front. The evolution of mixing rates is controlled by the competition between the advective stretching mechanism and such lateral compositional diffusion^12^. Following the onset of the previous regime, fast *advective* downward transport of fingers exceeds the *diffusive* rate of CO_2_ dissolution at the top. This depletion of CO_2_ concentrations in the fingers causes Δ*ρ*_max_ to drop soon after the fingering onset (Fig. 5). As the driving force for advection/buoyancy (Δ*ρ*_max_) weakens, an inflection point is reached where diffusion exceeds advection and stretching stops. The inflection point corresponds to the local minimum of Δ*ρ*_max_ and to the onset of a diffusion-dominated *restoration* regime (Figs 4d and 5), after which the dispersion-width returns to diffusive scaling as well (Fig. 4b). This third regime is comparable to a stagnation point in the advective flow where the rate of compression balances the rate of diffusive expansion^49^. During this transition period, CO_2_ transport is slower, concentrations are *replenished* (owing to the constant injection rate), and Δ*ρ*_max_ increases, similar to the first regime, until the dissolution front becomes gravitationally unstable once again.
4. The fourth regime is again convection-dominated and shows a sharp increase in the growth rates of the CO_2_ spatial variance and dispersion-widths. The duration of the first convective regime is shorter for high-permeability cases and the reduction in Δ*ρ*_max_ is smaller (Fig. 5). This regime occurs after 2 years for the 90% volume fraction in Fig. 3. This is in line with experimental results^17^ that show a reduced Δ*ρ* scaling as ~*Ra*^−0.2^ rather than a Rayleigh-independent constant dissolution flux^14^,^50^. Likewise, the third transition periods are shorter in high-permeability domains with less replenishment with CO_2_. Counterintuitively, this results in finger growth rates that are slower for the highest permeabilities (*σ*_*z*_ ∝ *t*^1.5^) than for the lowest permeability cases (*σ*_*z*_ ∝ *t*^4^) in this regime. This suggests that while Rayleigh and Péclet numbers are in the convection-dominated range for all permeabilities, they are lower for the high-permeability than for the low-permeability domains, due to the complex finger-brine interface at the end of the previous regime and the different depletion-replenishment histories of CO_2_ concentrations (and densities) inside the fingers.

### Heterogeneous media

In the previous section, we considered density-driven flow in homogeneous aquifers and found four alternating diffusion- and convection-dominated flow regimes, the extent of which depends on Rayleigh numbers. Next, we investigate whether similar behavior persists in realistic heterogeneous media that have regions of different facies with tortuous and potentially connected pathways. We expect that our global measures of CO_2_ spreading will be a mixture of the flow regimes observed for a range of homogeneous mean permeabilities. How the gravito-convective mixing of dissolved CO_2_ is controlled by realistic permeability fields may dramatically affect the long-term CO_2_ storage efficiency.

Comparing Figs 2 and 3 shows that fingering is more pronounced in the heterogeneous media. This is different from flow in unimodal heterogeneous media, characterized by (pressure-driven) permeability-channeling that dominates (gravity-driven) hydrodynamic fingering. The reason for this is the spatial organization of facies: for a given *mean* permeability, the corresponding bimodal facies distribution has both regions with much higher and with much lower permeabilities. In large connected regions of the high-permeability facies, the critical time and wavelength are shorter than those for the mean permeability, and fingering is more pronounced. In addition, small-scale fingers may survive longer without merging because they are shielded from each other by low-permeability facies.

In terms of our quantitative measures, Fig. 4a exhibits a higher degree of CO_2_ spreading throughout the domain at early times in heterogeneous compared to homogeneous cases. At later times (during the fourth regime in homogeneous domains), we observe a *crossover* after which CO_2_ spreading in heterogeneous media is no longer higher than that in homogeneous domains. The reason is that for all heterogeneous cases the onset of convective fingering (first deviation from Fickian scaling: *σ*_*z*_ ∝ *t*^*β*>0.5^) is almost instant, leading to more initial spreading but with a lower growth rate than for the homogeneous cases (Fig. 4c). Higher initial spreading ends in an earlier *convective-shutdown* regime in bimodal heterogeneities once the fingers reach the lower boundary and the domain starts to be saturated with CO_2_. This phenomenon has been studied in both numerical simulations and experiments as a late-time reduction of mixing rates^12^,^51^.

At late times, there is a *delay* in reaching the maximum spreading and a slower growth of *σ*_*z*_ for heterogeneous domains. This sub-convective behavior is caused partly by slow diffusion of CO_2_ into isolated low-permeability *islands* that locally impede the vertical propagation of descending fingers. More tortuous flow-paths can also be a factor, resulting in either a longer transit time of CO_2_-rich brine or favorable channeling of flow depending on the facies structure. *Blockage* of flow may also happen in bimodal structures, especially in aquifers with lower volume fractions of high-permeability facies.

The global measure of CO_2_ spreading is a superposition of fast convective (albeit tortuous) flow of CO_2_-rich fingers through high-permeability pathways and slower diffusive transport of CO_2_ through the lower-permeability facies. The former explains the higher initial mixing, while the latter causes the delay in reaching the maximum spreading. This phenomenon can be clearly seen in the 30% case (Figs 2 and 4a). For larger high-permeability volume fractions (above 50%), the delay in the spatial variance of CO_2_ over time is reduced. This is because we exceed the *percolation threshold* above which high-permeability clusters form large and connected preferential pathways that span the full extent of the domain^52^.

Scaling of the dispersion-width with time in Fig. 4c provides further insight into the complex flow dynamics of bimodal architectures. The four distinct flow regimes observed in homogeneous domains are smoothed out in heterogeneous formations. We find that CO_2_ transport in architectures below the percolation threshold scales from a diffusion-dominated Fickian regime (~*t*^0.5^) at early time, associated with flow through low-permeability facies, to a subsequent *ballistic* regime (~*t*) within high-permeability facies, and eventually to a *sub-diffusive* non-Fickian regime (~*t*^*β*<0.5^). The latter is due to a contribution from diffusive transport through low-permeability facies in a subset of the domain at late times. The ballistic regime is due to advective flow through connected high-permeability pathways with a linear growth rate of fingers^13^. Similar regimes of dispersion-widths have been observed in *viscously* unstable solute transport through heterogeneous porous media, in which flow starts ballistically and reaches a Fickian scaling in the late-time asymptotic stage, but showing no sub-diffusive spreading^48^,^53^.

We note that for facies architectures below the percolation threshold, the chronological order of Fickian and ballistic flow regimes are realization dependent: when high-permeability facies are concentrated at top of the domain, a ballistic period is likely to precede a Fickian diffusive regime and vice versa. Above the percolation threshold, we see predominantly ballistic flow through fully connected clusters, followed by sub-diffusive scaling in a final delay period that is shorter than that below the percolation threshold. For the high-permeability heterogeneous simulations, *σ*_*z*_ reaches the maximum spreading at comparable times as the equivalent homogeneous cases, because its temporal scaling is similar (*t*^1^ versus *t*^1.5^).

**Figure 6:**
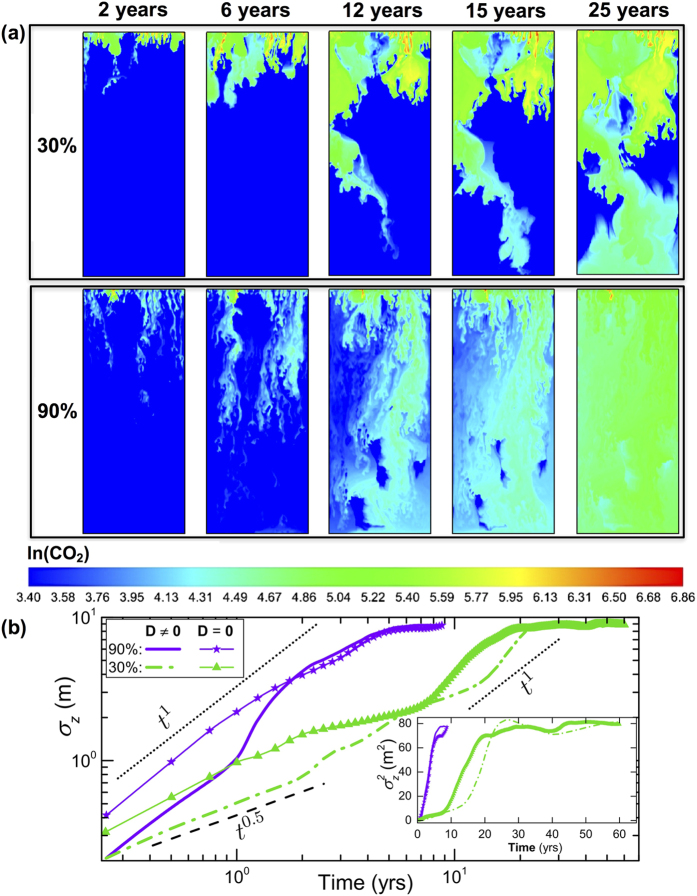
**(a)** Natural log of molar density of CO_2_ for simulations without Fickian diffusion for two *heterogeneous* cases with volume fractions of 30% and 90% of the high-permeability facies. **(b)** Dispersion-width for simulations with and without Fickian diffusion. Inset: temporal evolution of spatial variance for the same problems in linear scale.

We also model gravitational fingering in heterogeneous media at infinite Rayleigh numbers (*D* = 0) to isolate the predominantly advective flow (channeling) through high-permeability facies (for 30% and 90% volume fractions). Diffusive effects are generally more pronounced in heterogeneous (particularly, layered or fractured) aquifers in which compositional gradients tend to occur across interfaces between different permeabilities^54^. Expectedly, in the absence of diffusion as a restoring force, we see more dramatic fingering throughout the high-permeability facies (Fig. 6a). Our quantitative spreading measures show correspondingly higher values of *σ*_*z*_ and 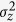 in Fig. 6b. Comparing the evolution of the spatial variance and dispersion-widths for simulations with and without diffusion, we see that diffusion has little impact on the global mixing rates in the 90% case because the flow is advection-dominated from the start. For the 30% case, diffusion causes more stagnation at early times and mainly delays the onset of ballistic regime. This suggests that while bimodal formations with low proportions of high-permeability facies may show less *vertical* spreading of CO_2_-rich brine, they can promote more *lateral* mixing due to diffusion into low-permeability zones at early times compared to those with higher volume fractions of such facies. For both the 30% and 90% facies distributions, maximum spreading is achieved at similar times with or without diffusion, confirming that the transport of CO_2_ is ultimately driven by advection-dominated, unstable, gravito-convective mixing.

**Figure 7:**
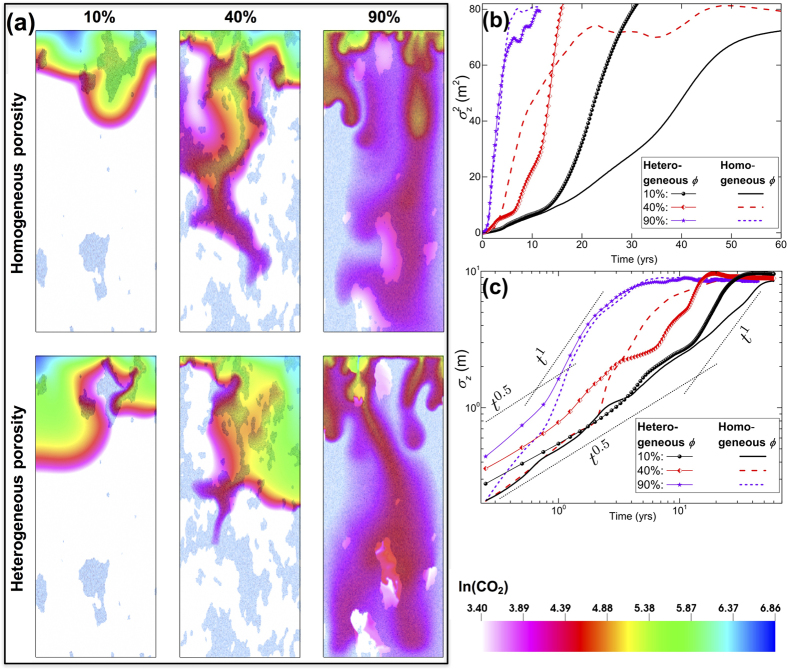
**(a)** Natural log of molar density of CO_2_ for simulations with homogeneous and heterogeneous porosity for three cases with volume fractions of 10%, 40%, and 90% of the high-permeability facies. Low-permeability facies are illustrated in white. **(b)** Spatial variance of molar density of CO_2_ (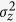). **(c)** Dispersion-width of molar density of CO_2_ (*σ*_*z*_).

Finally we study the impact of heterogeneous *porosity* distributions on density-driven CO_2_ mixing (Fig. 7). We select one realization each of the 10%, 40%, and 90% volume fractions. A ratio of 10 is assigned between the porosities of the two facies while keeping the average porosity at 10% to allow comparison to homogeneous media and to keep the injection rates the same. This means that the bimodal porosities for the low- and high-permeability facies are 5.3% and 53% for the 10% case, 2.7% and 27% for the 40% case, and 1% and 10% for the 90% case. Lower porosities formally reduce *λ*_*c*_, *t*_*c*_, and the diffusive flux (see Eq. (4)) and also affect CO_2_ transport (Eq. (3)) and its spatial variance (Eq. (7)). Lower permeability-porosity facies become saturated with CO_2_ earlier on, which increases the density contrast and triggers instabilities. As a result, while the advective flux is lower in low-permeability facies, the finger growth rates can be comparable to that in the higher permeability regions. This effect becomes more pronounced as the volume fraction of low-permeability facies increases. Figure 7b,c compare the temporal evolution of 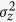 and *σ*_*z*_ for these cases. We observe a diffusion-dominated initial regime, followed by a ballistic regime for all bimodal porosity distributions, with flow in the low porosity facies more unstable than in the corresponding uniform porosities of 10%.

## Conclusions

We perform physically robust simulations of gravito-convective mixing of CO_2_ in saline aquifers without the limiting assumptions of prior studies. CO_2_ is injected into a confined domain and phase behavior is described by the cubic-plus-association EOS. CPA provides accurate densities, and accounts for both volume swelling due to CO_2_-dissolution, and compressibility due to injection-induced pressure increases. Heterogeneity, typical of fluvial deposits, is represented by bimodal facies architectures (e.g., shale and sandstone). To *quantify* the spreading of dissolved CO_2_ during the complex gravito-convective mixing, we evaluate global measures for the spatial variance and dispersion-width of CO_2_ as well as the evolution of a dispersion coefficient, the maximum density contrast Δ*ρ*_max_, and the velocity of the fastest growing unstable finger. From these measures, new flow regimes emerge at all Rayleigh numbers, caused by changes in Δ*ρ*_max_, the driving force for fast advective mixing. Due to competing diffusive and advective processes, we find in homogeneous media that for a constant dissolution rate in the top, Δ*ρ*_max_ initially scales as k^−0.5^. Moreover, the critical onset time of fingering scales as k^−1^, rather than the k^−2^ found from stability analyses that assume a constant Δ*ρ*_max_. Topological structures of sedimentary facies and their connectivity are responsible for additional complexities. Fingering is enhanced in connected high-permeability facies (especially above the percolation threshold). Low-permeability regions can delay downward advective transport, but diffusion through such regions results in a longer mixing tail. Solubility trapping is an important process in predicting the fate of injected CO_2_ in feasibility studies of carbon storage, and the subsurface distribution of CO_2_ is critical when evaluating, e.g., the risk of leakage through nearby abandoned wells. A promising result is that in bimodal facies architectures, convective mixing of dissolved CO_2_ starts much earlier leading to higher degrees of mixing than predictions for equivalent homogeneous media. Our detailed analyses of the scaling relations of various global measures of the flow dynamics can be used to upscale the computationally expensive fine-grid simulations to the large dimensions of saline aquifers.

## Methods

### Governing equations: species transport and pressure equations

We consider a fluid mixture consisting of 2 components, labeled by index *i* with *i* = 1 for brine and *i* = 2 for CO_2_. The advection-diffusion transport equation is represented in terms of each component’s molar density (*cz*_*i*_) as follows:


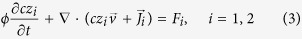


where *c* is the molar density, *z*_*i*_ the mole fraction of component *i* with *z*_1_ + *z*_2_ = 1, *F*_*i*_ [mol/m^3^s] a source term of component *i*, *ϕ* the porosity, *t* the time, 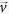 the advective flux and 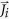 the diffusive flux, given by^55^:


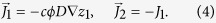


Velocities follow from Darcy’s law as:


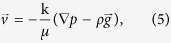


with *ρ*[kg/m^3^] the brine mass density, which is related to the molar density through the molecular weight of each component, *M*_*i*_, as *ρ* = *c*(*M*_1_*z*_1_ + *M*_2_*z*_2_).

Most studies of carbon sequestration assume an incompressible aqueous phase, while we allow for both fluid compression due to the pressure build-up during CO_2_ injection into an aquifer with impermeable boundaries, and volume swelling of brine due to CO_2_ dissolution. The pressure response is computed from^56^:


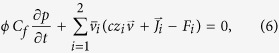


where *C*_*f*_ is the brine compressibility and 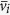 is the total partial molar volume of each component in the mixture.

### Numerical framework: higher-order finite element methods for flow and transport

We use numerical methods presented in earlier works^1^,^18^,^55^,^57^. A second-order discontinuous Galerkin (DG) method is used to update the transport equations. This approach has been demonstrated to reliably capture the small-scale onset of viscous and gravitational fingers on relatively coarse grids by reducing numerical dispersion^54^. Shahraeeni *et al*.^18^ showed that the higher-order mass transport update can resolve the critical wavelength with only a few elements. A Mixed Hybrid Finite Element (MHFE) method is used to solve the Darcy and pressure equations simultaneously, which provides accurate velocity and pressure fields. MHFE is particularly useful in the simulations presented in this article due to the sharp contrasts in the heterogeneous permeability fields.

Phase behavior is based on the cubic-plus-association (CPA) EOS. The CPA-EOS is suitable for water-containing mixtures and takes into account the hydrogen bonding of water molecules by thermodynamic perturbation theory. The physical interactions follow from the Peng-Robinson EOS. In addition, the CPA-EOS allows for *cross-association* of CO_2_ with water, which is induced by polar-polar interactions^1^,^35^,^36^. This approach is an improvement over previous studies that relied on Henry’s law for CO_2_ solubilities and empirical correlations for the brine density.

### Characterization of CO_2_ spreading: spatial variance and dispersion-width of CO_2_ molar density

In order to *quantify* the degree of mixing due to density-driven flow of CO_2_, we compute the spatial variance 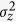 of the molar density of CO_2_ and the dispersion-width *σ*_*z*_^41^. The choice of CO_2_ molar density, rather than composition, accounts for both the density change due to CO_2_ dissolution and the compressibility of the aqueous phase.

The first three central moments of the distribution define the spatial variance 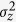 of the molar density of CO_2_ (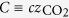):


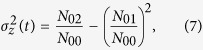


where *N*_01_/*N*_00_ expresses the coordinate location of the center of mole in the vertical (*z*) direction. The *N*_*ik*_ is the *ik*-th moment of the CO_2_ molar density distribution in space, analogous to the moment of concentration distribution^58^, defined here as:


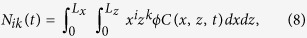


with *L*_*x*_ and *L*_*z*_ the domain size in *x*- and *z*-directions, respectively. The zeroth moment, *N*_00_, physically represents the total number of moles of CO_2_ in the domain. The *N*_00_ does not remain constant over time because CO_2_ is continuously injected into the domain, unlike previous studies involving a pulse input of a solute in solution^59^. However, by normalizing the first and second moments by *N*_00_, the spatial variance becomes insensitive to increasing *N*_00_. The variance in the *x*-direction is assumed to be negligible due to the dominant *downward* migration of CO_2_-rich brine. As a result, *i* equals to 0 in the *ik*-th moments.

While spreading and dispersion can fundamentally differ from mixing and dilution, especially in highly heterogeneous media^48^, it is a reasonable proxy to mixing, especially when there is a variable total volume of the dissolving solute in the system, because more spreading usually leads to more dilution and mixing^60^. The 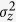 or *σ*_*z*_ reach their maximum values, above which they plateau, shortly after entering a convection-shutdown regime. During this period, it can be concluded that spatial fluctuations of CO_2_ concentration about its mean value will gradually vanish as CO_2_ begins to saturate the entire domain. This implies a mixing degree that will finally increase to unity at the *perfect* mixing state, though with dissipative rates. To obtain an estimate of the maximum value of 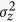, we can assume a constant and spatially invariant non-zero *C*(*x*, *z*, *t*) throughout the domain and analytically solve the integral in Eq. (8). This results in 
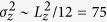
 m^2^, i.e. close to the values observed in Fig. 4a (higher values are partly due to compressibility as the pressure increases).

## Additional Information

**How to cite this article**: Soltanian, M. R. *et al*. Critical Dynamics of Gravito-Convective Mixing in Geological Carbon Sequestration. *Sci. Rep.* **6**, 35921; doi: 10.1038/srep35921 (2016).

**Publisher’s note**: Springer Nature remains neutral with regard to jurisdictional claims in published maps and institutional affiliations.

## Figures and Tables

**Table 1:** Geometric mean permeability and critical time for homogeneous media.

Volume fraction (%)	Geometric mean (md)	Critical time (years)			
		Slim and Ramakrishan^46^	Riaz *et al*.^13^	Pau *et al*.^14^	This study
10	147.86	0.16462	5.017	3.96–12.61	17.25
20	218.36	0.07548	2.301	1.82–5.78	11.50
30	324.12	0.03426	1.045	0.826–2.62	8.50
40	479.02	0.01568	0.478	0.378–1.20	5.25
50	707.92	0.00718	0.223	0.173–0.55	3.25
60	1044.49	0.00330	0.101	0.0795–0.25	2.25
70	1544.53	0.00151	0.046	0.0364–0.115	1.50
80	2286.82	0.00069	0.021	0.0166–0.053	1.00
90	3381.09	0.00031	0.009	0.0076–0.024	0.75

The geometric mean of permeability increases for larger volume fractions of high-permeability facies.
